# Supplementary figures and images for: The Multi-Kinase Inhibitor EC-70124 Is a Promising Candidate for the Treatment of FLT3-ITD-Positive Acute Myeloid Leukemia
Source: Cancers (Basel). 2022 Mar 21;14(6):1593. doi: 10.3390/cancers14061593 (PMC8946166; doi:10.3390/cancers14061593)

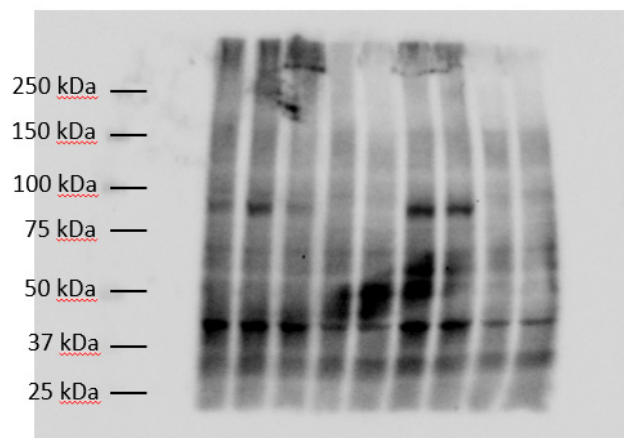

**pStat**

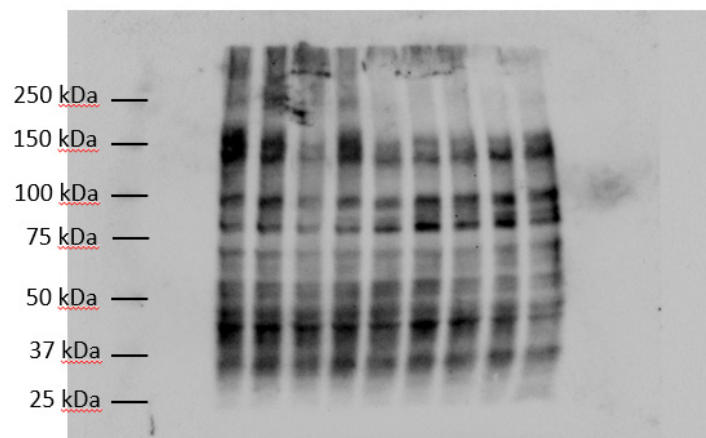

**Stat**

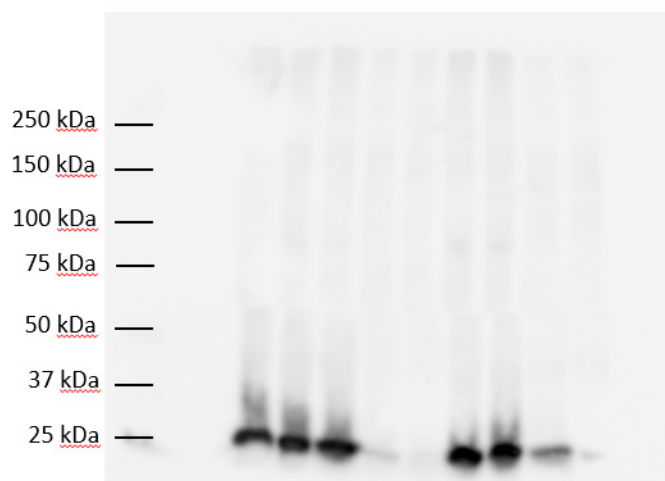

**pS6**

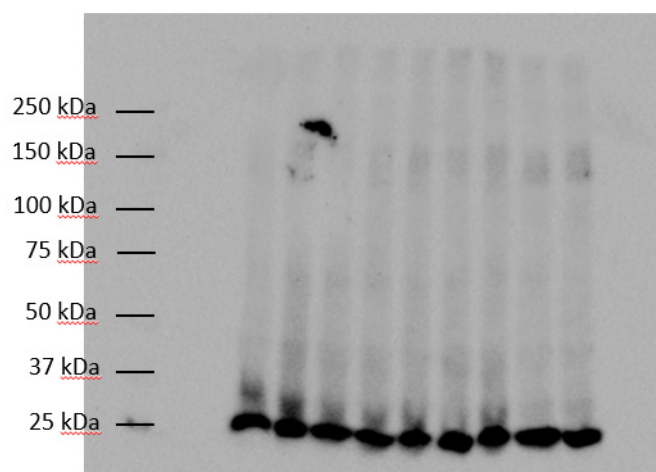

**S6**

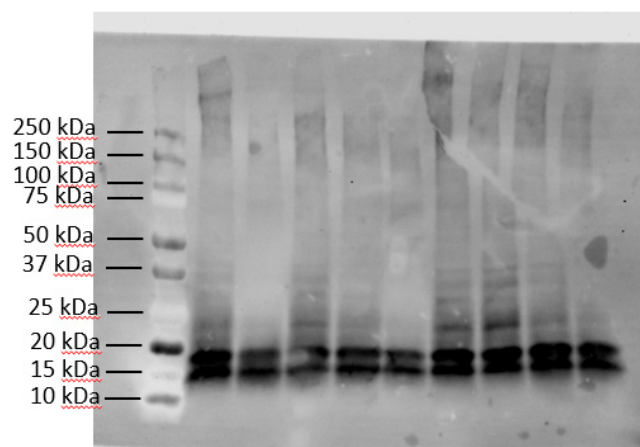

**pBad**

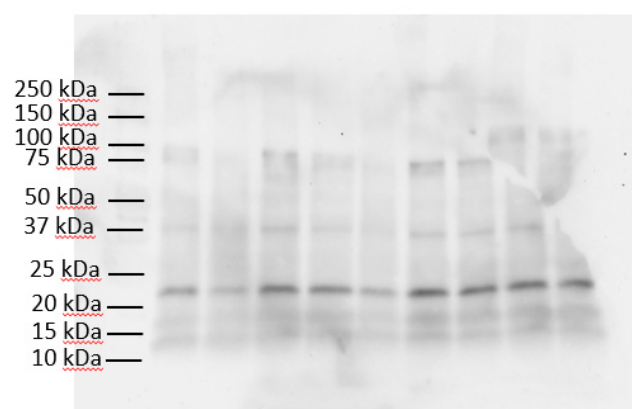

**Bad**

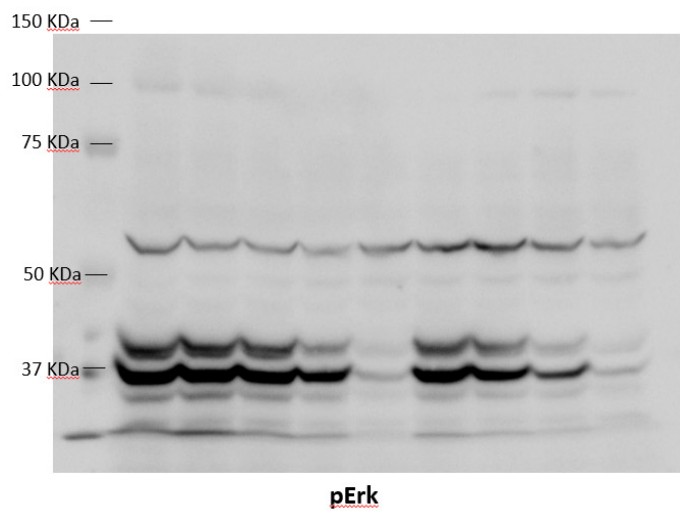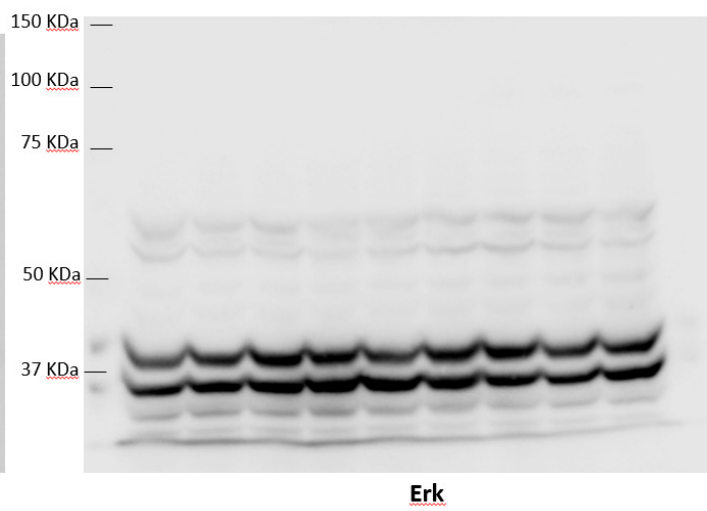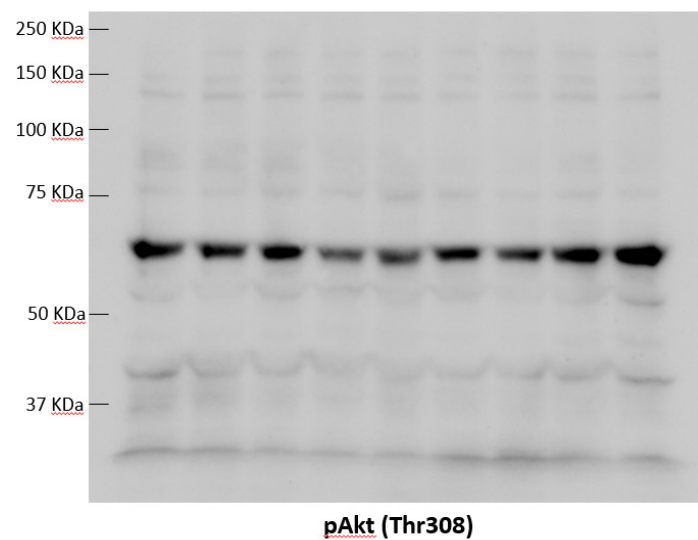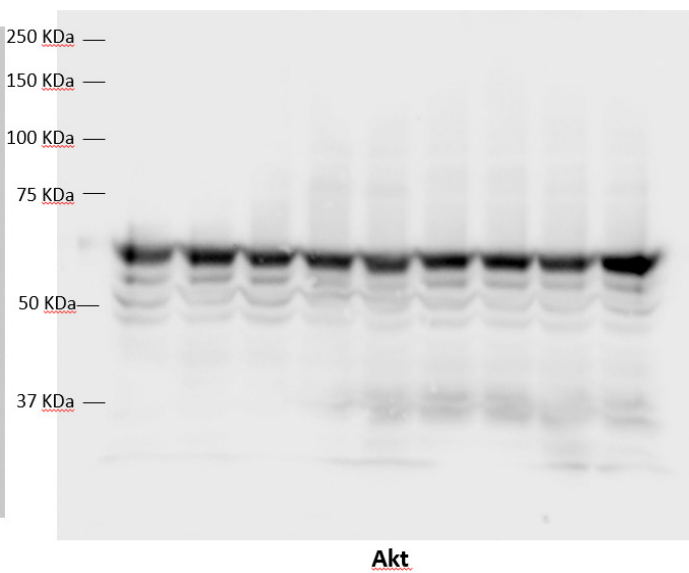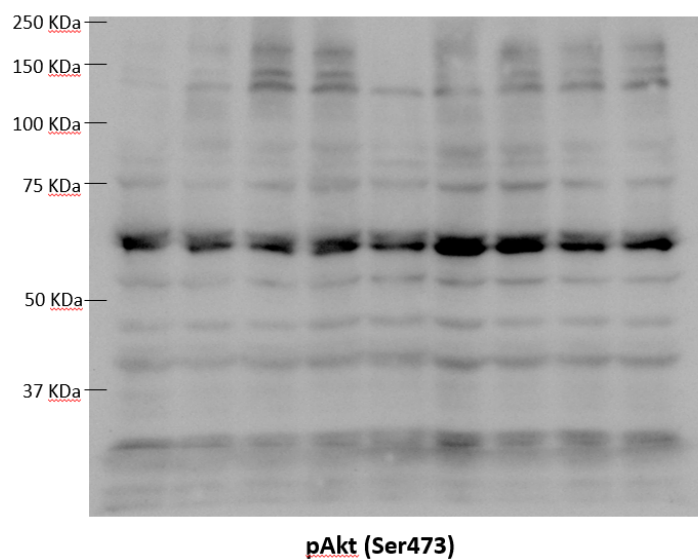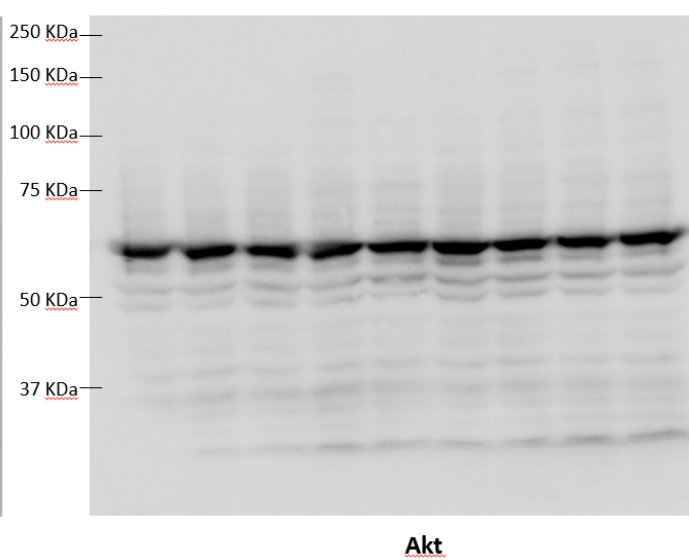

Figure S1. Uncropped blots.

Supplement: Supplementary file 1 [file cancers-14-01593-s001.zip › cancers-1643669-SI.pdf]
